# The footprint of urban heat island effect in China

**DOI:** 10.1038/srep11160

**Published:** 2015-06-10

**Authors:** Decheng Zhou, Shuqing Zhao, Liangxia Zhang, Ge Sun, Yongqiang Liu

**Affiliations:** 1 Jiangsu Key Laboratory of Agricultural Meteorology, and College of Applied Meteorology, Nanjing University of Information Science and Technology, Nanjing 210044, China; 2 College of Urban and Environmental Sciences, and Key Laboratory for Earth Surface Processes of the Ministry of Education, Peking University, Beijing 100871, China; 3 Eastern Forest Environmental Threat Assessment Center, Southern Research Station, USDA Forest Service, Raleigh, NC 27606, USA; 4 Center for Forest Disturbance Science, Southern Research Station, USDA Forest Service, Athens, GA 30602, USA

## Abstract

Urban heat island (UHI) is one major anthropogenic modification to the Earth system that transcends its physical boundary. Using MODIS data from 2003 to 2012, we showed that the UHI effect decayed exponentially toward rural areas for majority of the 32 Chinese cities. We found an obvious urban/rural temperature “cliff”, and estimated that the footprint of UHI effect (FP, including urban area) was 2.3 and 3.9 times of urban size for the day and night, respectively, with large spatiotemporal heterogeneities. We further revealed that ignoring the FP may underestimate the UHI intensity in most cases and even alter the direction of UHI estimates for few cities. Our results provide new insights to the characteristics of UHI effect and emphasize the necessity of considering city- and time-specific FP when assessing the urbanization effects on local climate.

Urbanization, one major anthropogenic modification to the Earth system, is accelerating at an unprecedented rate in recorded human history worldwide^1^,^2^. More than half of world’s population live in urban areas now, and this number is projected to be 67% by 2030^3^. To meet the needs of soaring city dwellers, global urban land is now expanding at twice the population growth rate^1^ and is expected to nearly triple the area in circa 2000 by 2030 provided with current population density^4^.

Urbanization can pose many negative impacts on Earth’s environments that transcend far from its physical boundary^5^. Among these impacts, the urban heat island (UHI), referred as the phenomenon that urban areas tend to have higher temperatures than surrounding areas, has long gained considerable interest among scientists and urban planners^6^,^7^,^8^,^9^,^10^,^11^,^12^. UHI has many potential impacts on water and air quality, microclimatology, vegetation growth^5^,^9^,^13^, and human health (e.g., increase in morbidity and mortality)^5^,^14^,^15^,^16^. The UHI effects have been observed in both urban and adjacent suburban areas^17^,^18^. Thus, there is a strong impetus to systematically understand the UHI not only in its magnitude (UHI intensity, UHII) but also in its extent (also referred as the footprint of UHI effect in this study, FP).

UHII is loosely defined as the temperature difference between urban and surrounding areas. Due to the poor knowledge of the FP, estimates of UHII can vary dramatically^19^. For example, the UHII defined as the temperature difference between urban area and suburb^10^,^11^,^20^ might be lower than that between urban and rural areas^17^,^21^. Unfortunately, to date nearly all the UHI studies used the areas with static and/or subjective distances away from the urban perimeters as the unaffected references^10^,^11^,^20^,^22^,^23^, resulting in large bias in the UHII estimates and difficulty to compare among different studies. Better knowledge of the FP therefore can help not only for better understanding of the UHI phenomenon, but also is essential for an accurate estimate of UHII.

However, a systematic evaluation of the FP over large areas is still lacking. To our knowledge, Zhang *et al.*^18^ might be the only study that addressed this issue at a regional level. They found that the FP on average can reach up to 2.4 times of the actual urban land cover (including urban area) in the eastern North America, but they did not explore the spatiotemporal variability. The most recent global study^10^ also indicated that the UHI effect was mainly limited within the area twice the urban area, but it did not quantify the FP explicitly. Numerous studies documented that the UHII varied substantially across space and time^10^,^11^,^17^,^20^,^21^,^22^,^23^, suggesting that the FP might also have a great spatiotemporal variability. A comprehensive study on the FP is thus needed across diverse cities to understand the spatial patterns and controlling factors.

In this study, we examined the UHI effect in 32 major cities distributed in different climatic zones of China (Fig. 1) using Moderate Resolution Imaging Spectroradiometer (MODIS) Land Surface Temperature (LST) products (version 5) in conjunction with cloud-free Landsat Thematic Mapper (TM) and Enhanced Thematic Mapper Plus (ETM+) images during the period from 2003 to 2012. Unlike most previous efforts that focused on UHII^10^,^11^,^21^,^22^, we mainly concentrated on the FP of those cities. China is ideal to investigate the FP at a regional level, since it has been experiencing the rapidest urbanization in the world in recent decades^2^,^3^,^4^ and is characterized by complex zonal variations (from the tropical to subarctic/alpine and from rain forest to desert). Our main objectives were to (1) investigate the trends of UHI effect along urban-rural gradients (Fig. 2), (2) explore the spatiotemporal variability of the FP, and (3) examine the possible UHII bias induced by ignoring the FP by comparing urban-suburban and urban-rural LST differences for those 32 major cities across China. Twelve buffers surrounded urban areas were generated for each city (Fig. 2) and the UHI effect (△T) in urban and nearby buffer zones were defined as their LST differences relative to unaffected rural reference (see Methods). The FP was defined as the continuous extent emanating outward from urban centers to rural areas that have evident UHI effect (i.e., △T was statistically larger than zero).

## Results

### Exponential decay of UHI from the urban area

The annual mean daytime UHI effect (△T) decreased exponentially and significantly (*p* < 0.05) toward rural areas for 29 of 32 cities, except for Lanzhou, Lhasa, and Tianjin (Fig. 3, Supplementary Table S1). For all cities combined, the △T decayed exponentially as a function of distance (*d*) with the maximum temperature difference (*A*) of 1.78 ± 0.08 and the decay rate (*S*) of 1.30 ± 0.12, respectively (the formula takes the form of *△T = A* × e^*−S×d*^ + *T*_0_, *r*^*2*^ = 0.98, *p* < 0.01, Fig. 4A). Comparatively, the annual mean night time △T declined exponentially (*p* < 0.05) with distance for all the 32 cities (Fig. 5, Supplementary Table S1), with overall *A* and *S* of 1.59 ± 0.02 and 0.71 ± 0.03, respectively (*r*^*2*^ = 1.00, *p* < 0.01, Fig. 4B). In addition, the daytime △T decreased more sharply in the summer than in the winter period (Figs. 3 and 4). Specifically, all cities except for Lanzhou exhibited significant decreases in summer, whereas over three quarters of the cities had insignificant trends in winter (Supplementary Table S1). On average, the *A* was 2.83 ± 0.06 in summer, significantly larger than that in winter (0.18 ± 0.04) (Fig. 4A). By contrast, the △T trends differed slightly by season at night (Figs. 4 and 5, Supplementary Table S1). Note that an evident cold island effect during the day was observed in both summer and winter for Lanzhou and in winter for Tianjin (Fig. 3).

### The footprint of UHI effect

The footprint of UHI effect (FP) varied substantially by cities and time periods (Fig. 6). On a yearly basis, the △T was evident for 29 of 32 cities with an exception of Hohhot, Lanzhou (i.e., cold island effect), and Tianjin in the daytime, with FP ranging from 1.0 (Hangzhou and Lhasa) to 5.5 (Shijiazhuang) times of actual urban size. The night △T was obvious for 31 of 32 cities but Nanning, with the FP fluctuating between 1.0 (Guangzhou) and 6.5 (Harbin, Urumqi, and Taiyuan). Overall, the FP at night reached up to 3.9 times of urban area and was significantly larger than that in the day (2.3) (*p* < 0.01, Paired-Samples T Test, Fig. 7). If we transformed the FP into physical distance away from urban edge, the annual mean FPs in the day and night amounted to 3.4 and 5.7 km away from urban perimeters, respectively. Furthermore, the daytime FPs in general were larger in the summer than the winter period (2.8 vs. 1.2 times of urban area, *p* < 0.01), whereas the night time FPs were similar in both seasons (3.6 vs. 3.5, *p* = 0.69) (Fig. 7). In particular, many cities had no significant UHI effect during the day in winter (Fig. 6).

The spatial distribution of the FPs (unit: times of urban area) related significantly and positively to UHII across cities (Table 1), with the strongest relationship at night during winter (*r* = 0.74, *p* < 0.01). Also, it linked negatively to background air temperature and precipitation in the day for summer and at night for both summer and winter, while the opposite relationships were observed in the day for winter. If we converted the FP into physical area (unit: km^2^), the FP correlated tightly and positively with actual urban size, with the correlation coefficients higher than 0.79 (Fig. 8).

### Comparison of urban-rural and urban-suburban LST differences

The varying FPs by cities and time periods suggested that methods ignoring the FP might result in large bias in UHII estimates. To test this, we compared the UHIIs under two most commonly used definitions: urban-rural (assumed no UHI effect in the rural area and it can represent the control) and urban-suburban (assumed there is UHI effect in suburban area, accompanied by the bias of UHII estimates) LST differences for those 32 cities. The LST differences between urban and rural areas were significantly larger than that between urban and suburban areas during the day and night for nearly all the cities, except for Lanzhou and Tianjin in the day (Fig. 9). The annual mean urban-rural LST differences were 1.7 ± 1.2 ^o^C in the day and 1.6 ± 0.7 ^o^C at night, which were 1.8 and 2.4 times that of urban-suburban LST differences in the day (0.9 ± 0.5 ^o^C) and night (0.7 ± 0.4 ^o^C), respectively. Particularly, Lanzhou presented evident cold island effect if compared to rural area (−2.7 ^o^C), but weak heat island effect relative to nearby suburb (0.2 ^o^C) during the day. In contrast, Xining witnessed weak heat (0.4 ^o^C) and cold (−0.2 ^o^C) island effect during the day compared to rural and suburban areas, respectively, and similar phenomenon happened in Chongqing at night. Overall, however, there were strong linear relationships between these two kinds of estimates across cities during the day (*r* = 0.70, *p* < 0.01) and night (*r* = 0.71, *p* < 0.01).

## Discussion

### Exponential decay of the UHI effect along urban-rural gradients

We showed that the UHI effect decayed exponentially and significantly with distance away from urban areas for majority of 32 Chinese cities, with the maximum temperature in the urban center and a temperature “cliff” between urban and rural areas (Figs. 4). This was similar to the findings in the United States^17^,^18^. The decline trends were observed more obvious in summer than winter during the day, which can be mainly attributed to much stronger UHI effect^10^,^11^ and much more active convection together with the related circulation^24^ in summer than winter during the daytime. On annual basis, although the maximum temperature difference were similar for the day and night (1.78 vs 1.59), the decay rate in the day was nearly twice that at night (1.30 vs 0.71), suggesting that the FP was much smaller in the day than at night as discussed further in following section.

Exceptions to above-mentioned patterns were also observed. One arid land-locked city (i.e., Lanzhou) exhibited an evident temperature decline trend, the “cold island effect”, along urban-rural gradients in the day during summer (Fig. 3). This is possibly due to the cooling effect (more heat being used for evapotranspiration) induced by the relatively higher vegetation activity and soil moisture (by irrigation) in urban and nearby buffer zones compared to rural areas^25^. Moreover, two cities (Lanzhou and Tianjin) presented obvious decay trends of cold island effect toward rural areas during the day in winter. The reason for this phenomenon was not clear, but might be related to the air pollution^11^ and vegetation activity in this season. Heavy air pollution in winter for the northern cities resulted from large amount of coal burned for heating^26^ can decrease incoming short wave solar radiation in the urban area compared to the rural area^27^, which in turn might artificially reduce urban temperature and even result in cold island effect. Low vegetation activity in winter exerted little cooling effects on rural surface, where usually has more available energy than its urban counterpart during the day^28^, therefore might result in urban cold island in those cities as well.

### Spatiotemporal variability of the FP across cities

The footprint of UHI effect (FP) varied dramatically by cities (Fig. 6). As expected, the FP was larger provided with higher UHII and/or actual urban size, in particular at night (Table 1, Fig. 8). For example, the higher nighttime FPs in Northern China can be mainly attributed to the significant higher UHII in the region compared to southern cities^11^. At the same time, the background climate influenced the FP substantially (Table 1), due to the climate control on the UHII^11^,^12^ and many thermodynamic properties over large areas^7^,^8^. Larger FPs happened in colder or drier cities of China during the night in both summer and winter (Fig. 6), while the opposite occurred during the day in winter. These observations can be largely explained by the climate-driven UHII distribution in those time periods^11^. Unexpectedly, smaller FPs were observed in some humid hot cities such as Guangzhou, Hangzhou and Nanjing during the day in summer (Fig. 6) although with relatively higher UHII^11^. This phenomenon was likely caused by land use patterns^29^. A strong contrast of land surface conditions (urban vs. vegetation) and thus energy balance^25^ can exaggerate the decay trends of UHI effect toward rural areas, and therefore dwarf the FPs. For example, rice paddy fields or other wetlands with no water limitation surrounding urban areas can have much higher evapotranspiration rates than urban land, thus the cooling effects of rural lands can sharply drop the daytime land surface temperature^30^. Further, other factors such as built-up intensity, topography, and landscape configuration may be also responsible for the FP’s variability. For example, the areas surrounded urban areas were usually covered by some built-up land. Higher built-up intensity in the area helps maintain warmer temperature^24^, consequently elevating the UHI effect and possible the FP of the city. Overall, the FP’s distribution appears to be more complex in the day than at night, most likely due to more complicated mechanisms underlying the UHI effect in the day than at night^11^.

The FP differed greatly by time periods. The average FP equals to 2.3 times of urban area in the day, comparable to the estimates (i.e., 2.4) by Zhang *et al.*^18^ in the eastern North American. Yet it was significantly less than the FP at night (i.e., 3.9). The day-night difference can be attributed to the varying mechanisms underlying the day and night UHI effects^7^,^9^,^10^,^11^. For example, vegetation activity, cooling the Earth surface via transpiration, was one major driver for daytime UHI effect^10^,^11^. Urbanization, replacing natural or semi-natural vegetation with impervious surfaces, often results in a sharp urban-rural temperature “cliff” in the day, therefore reduce the FP during the daytime. Comparatively, vegetation exerts weak effect on nighttime UHI because transpiration usually does not occur at night. At the same time, local convection and advection, suppressing the UHI and thus the FP, were much stronger during the day (particularly in summer) than at night^24^. Moreover, we found that the FP was larger in the summer than the winter during the day, most likely due to the overwhelmingly higher UHII in summer than winter during the day^11^. By contrast, the nighttime FP varied slightly by season, which can be attributed to the small seasonal changes of the UHII at night^10^,^11^.

### Possible UHII bias induced by ignoring the FP

Our results showed that the UHII, if defined as the LST differences between urban and unaffected rural areas, was significantly higher than that between urban and suburban areas for nearly all the cities, in particular at night (Fig. 9). This led us to conclude that the UHII will be most likely underestimated if the FP was neglected. Further, the direction of the UHII estimates might be reversed. For instance, Lanzhou presented cold island effect (negative) compared to rural areas but weak heat island effect (positive) relative to suburban areas during the day. This can be attributed to the higher vegetation activity (cooling effects during the day) in suburb, followed by urban zones compared to rural areas over the city^25^. In contrast, a weak heat island effect was observed in Xining compared to rural areas but cold island effect compared to suburb, possibly due to intensive agricultural practices in the rural areas, which led to higher vegetation activity compared with its urban and suburban counterparts^25^. These together highlight the importance of considering city-specific FP when assessing the urbanization effects on local climate. Interestingly, the spatial variation of the UHII across cities under the two definitions were closely related, possibly because the large-scale UHII’s distribution was overwhelmingly controlled by the background climate^11^,^12^,^17^, which differed slightly by how we define UHII for a city.

### Uncertainties

Uncertainties remained in the results from this analysis. First, the FP might be underestimated in a few cities at night, especially during winters. We analyzed the UHI effect in the area extent less than seven times of actual urban area (including urban area) and assumed that the LST in the three furthest buffers can be considered as the unaffected references. However, our results showed that the FP reached up to 6.5 times of urban area for few cities, which was already the unaffected references defined in this study, indicating that the FP for those cities might be larger if we expanded the study area. We did not extend the research area in order to reduce the uncertainties associated with topography, land use, and the coupling UHI effect with neighboring cities. Second, two cities demonstrated cold island effect in the day in winter. We speculated that air pollution and low vegetation activity might contribute to this phenomenon, and direct observations are needed to understand the underlying mechanisms. Moreover, as mentioned above, many factors not included in this paper may play important roles in the FP’s distribution. We did not explore them in detail owing to the data and technique limitations. These together stressed the necessity of more efforts on exploring the FP via the combination use of observation, remote sensing, and physical-based models^28^ in future works.

## Methods

### Datasets

**Figure 1:**
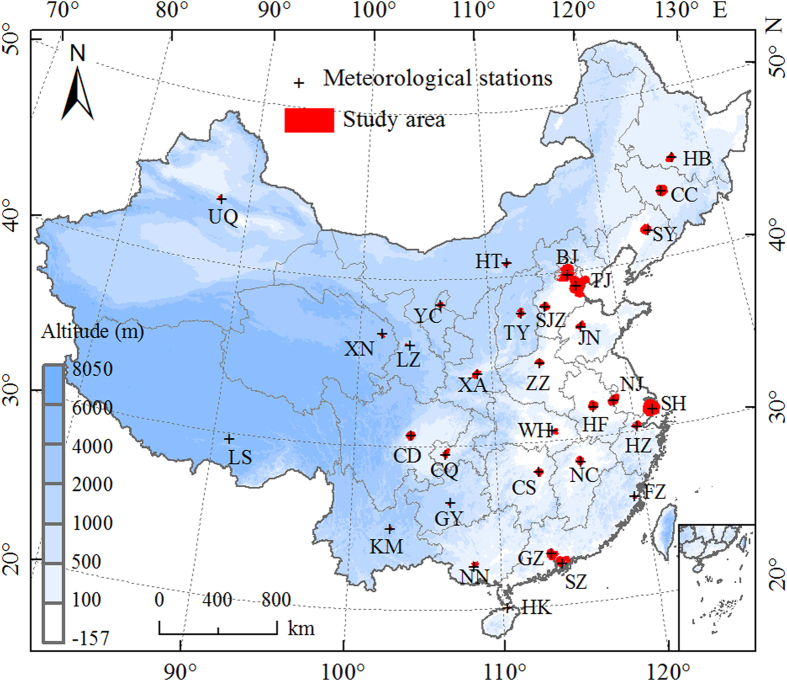
Locations of the 32 major cities in China. All of the cities are municipalities or provincial capitals except Shenzhen, which is China’s first special economic zone, and is now considered one of the fastest-growing cities in the world. The red areas on the map were included in this analysis, which excluded the altitude effects and water pixels. BJ: Beijing; CC: Changchun; CS: Changsha; CD: Chengdu; CQ: Chongqing; FZ: Fuzhou; GZ: Guangzhou; GY: Guiyang; HK: Haikou; HZ: Hangzhou; HB: Harbin; HF: Hefei; HT: Hohhot; JN: Jinan; KM: Kunming; LZ: Lanzhou; LS: Lhasa; NC: Nanchang; NJ: Nanjing; NN: Nanning; SH: Shanghai; SY: Shenyang; SZ: Shenzhen; SJZ: Shijiazhuang; TY: Taiyuan; TJ: Tianjin; UQ: Urumqi; WH: Wuhan; XA: Xi’an; XN: Xining; YC: Yinchuan; ZZ: Zhengzhou. Map was generated using ArcGIS 9.3 (www.esri.com/software/arcgis).

We focused on 32 major cities in China (Fig. 1), all of them are municipalities or provincial capitals except Shenzhen, which is China’s first special economic zone considered as one of the fastest growing cities in the world. Urban coverage maps of each city were obtained from our previous studies^11^,^25^, which were derived from the cloud-free Landsat Thematic Mapper (TM) and Enhanced Thematic Mapper Plus (ETM+) images (downloaded free from http://www.usgs.gov/ and http://datamirror.csdb.cn/) with a resolution of 30 m. The Landsat remote sensing data over 2004–2006 and 2009–2011 (Supplementary Table S2) were utilized to represent two time periods of circa 2005 and 2010, respectively. Details on land use classification can be found in Zhou *et al*^11^. Land surface temperature (i.e., LST) was obtained from Aqua MODIS 8-days composite products (version 5) with a spatial resolution of 1 km (MYD11A2) from 2003 to 2012. The LST data, including temperature observations that were monitored at 13:30 h (daytime) and 1:30 h (nighttime) local solar time, were estimated using a generalized split-window algorithm^31^. The retrieval of LST was further improved by correcting noise resulting from cloud contamination, topographic differences, and zenith angle changes, with the absolute bias generally less than 1 K^32^,^33^. Annual climate data of precipitation and air temperature from 2003 to 2012 were used to reflect the climatic background, which were obtained from Chinese Meteorological Observations (http://cdc.cma.gov.cn/). The meteorological station was located within urban area or nearby suburb for each city (Fig. 1). Digital Elevation Model (DEM) at a 3 arc-second (approximately 90 m) spatial resolution from the Space Shuttle Radar and Topography Mission (SRTM) (downloaded from http://earthexplorer.usgs.gov/) was also utilized in order to exclude altitude effects^25^.

### Analysis

In this study, we hypothesized that the UHI effect (i.e., △T) should be greatest in urban area and decrease gradually towards rural area^17^,^18^,^34^. To test this, it is necessary to define and delineate urban and surrounding buffer areas on maps. The urban area was defined by three steps^11^: 1) a built-up intensity (BI) map was generated from each urban coverage map using a 1 km × 1 km moving window method (to match the pixel size of MODIS LST data); 2) a 50% threshold of BI was used as a criterion^11^,^17^,^35^ to separate the BI maps into high- and low-intensity built-up land; and 3) the high-intensity built-up polygons were aggregated to delineate the urban border with an aggregation distance of 2 km, which is equivalent to two MODIS LST pixels and sufficient to include the scattered and most adjacent high-intensity built-up patches into the urban class. The land within the border was considered as urban area (excluding water pixels), which ranged from 47.6 km^2^ (Lhasa) to 2350.6 km^2^ (Tianjin) in year 2010.

**Figure 2:**
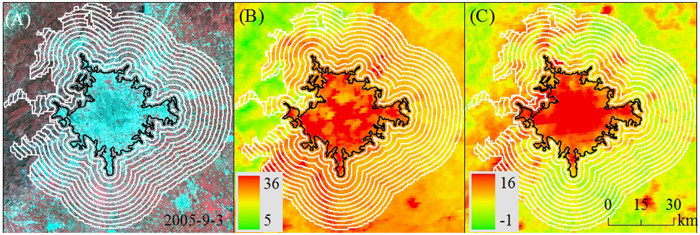
The delineation of urban area and twelve buffer zones, an example of Beijing. Landsat TM false color image acquired in Sep 3rd 2005 with a spatial resolution of 30 m × 30 m (**A**), daytime land surface temperature (LST) (**B**), and nighttime LST (**C**) in 2005. The black line stands for the border of urban area, the land within the border was considered as urban area, and the white lines represent the border of buffers (each of them covers half of actual urban area). Pixels that were water body or with elevation more than 50 m higher than the highest point in urban area were excluded from this analysis. Maps were generated using ArcGIS 9.3 (www.esri.com/software/arcgis).

**Figure 3:**
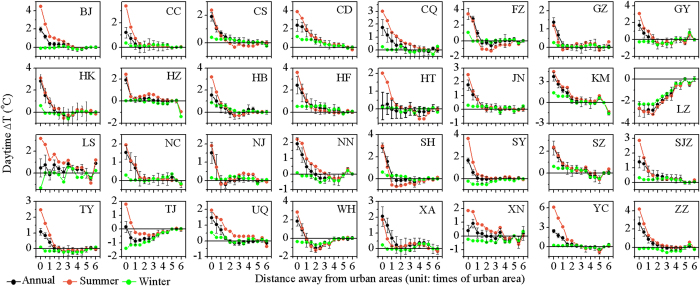
Trends of urban heat island effect (△T, defined as the LST differences relative to unaffected rural areas) from urban to rural areas during the day for China’s 32 major cities averaged over 2003–2012. The error bars represent the standard deviation.

**Figure 4:**
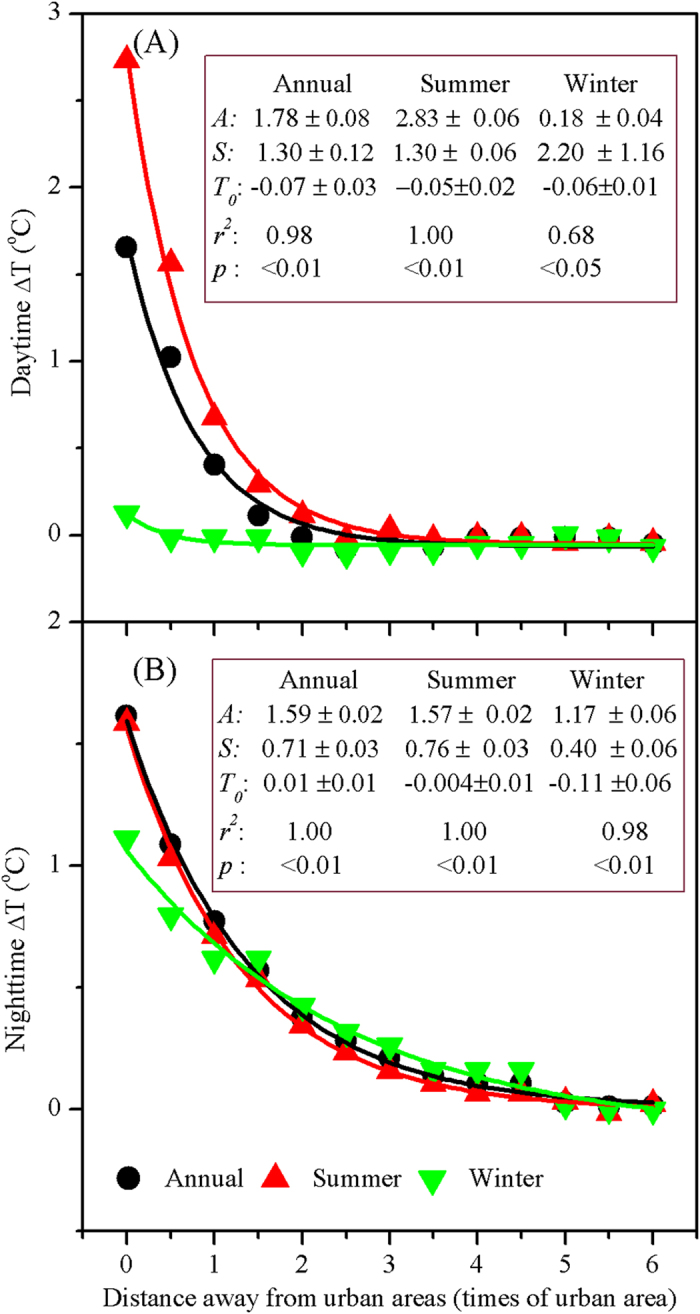
Exponential trends of the △T with distance (*d*) away from urban to rural areas for China’s 32 major cities averaged over 2003–2012. The function takes form of *△T* = *A* × e^*−S×d*^ + *T*_0_, where *A* indicates the maximum temperature difference, *S* is the decay rate, and *T*_*0*_ is the asymptotic value that the exponential trend can reach.

**Figure 5:**
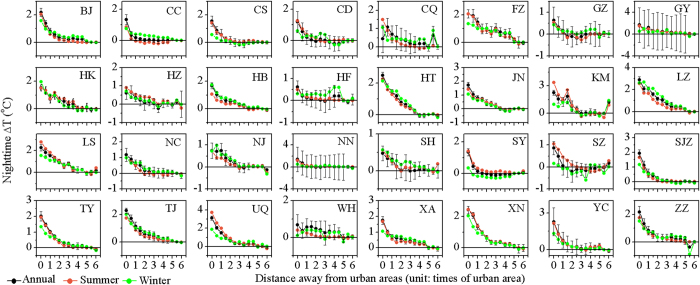
Trends of △T from urban to rural areas during the night for China’s 32 major cities averaged over 2003–2012. The error bars represent the standard deviation.

**Figure 6:**
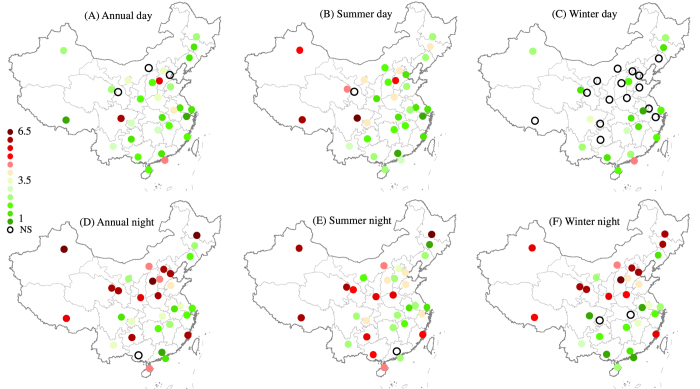
The footprint of urban heat island effect (FP, times of urban area) for China’s 32 major cities averaged over 2003–2012. The hollow black circle indicates no significant urban heat island effect for the city (NS). Maps were generated using ArcGIS 9.3 (www.esri.com/software/arcgis).

**Figure 7:**
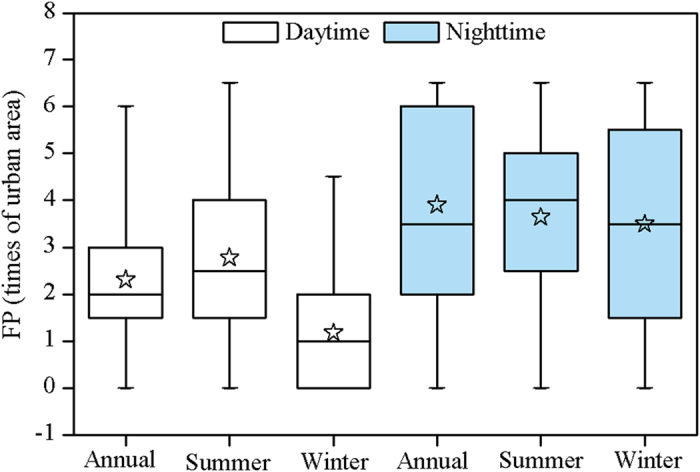
Box and whisker plots for annual, summer, and winter FPs during daytime and nighttime over China’s 32 major cities. The boxes represent the 25% to 75% range, the whiskers indicate the minimum and maximum values, and the open pentagrams demonstrates the mean values.

**Figure 8:**
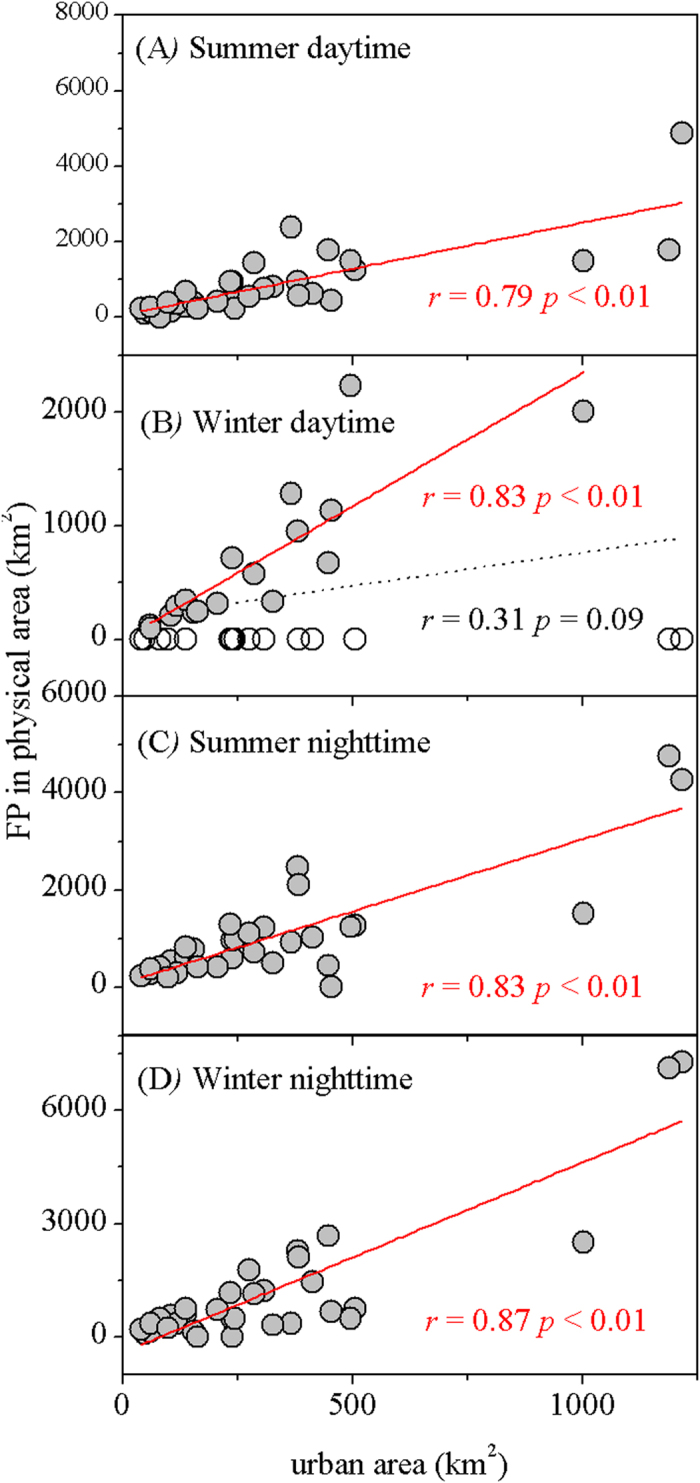
Relationship between the areas of the FP and actual urban land cover across China’s 32 major cities. The relationship was not significant during daytime in winter (panel B) because 15 of 32 cities have no UHI effect (shown as empty circle), but the correlation was significant at 0.01 level if we excluded those cities with no UHI effect.

**Figure 9:**
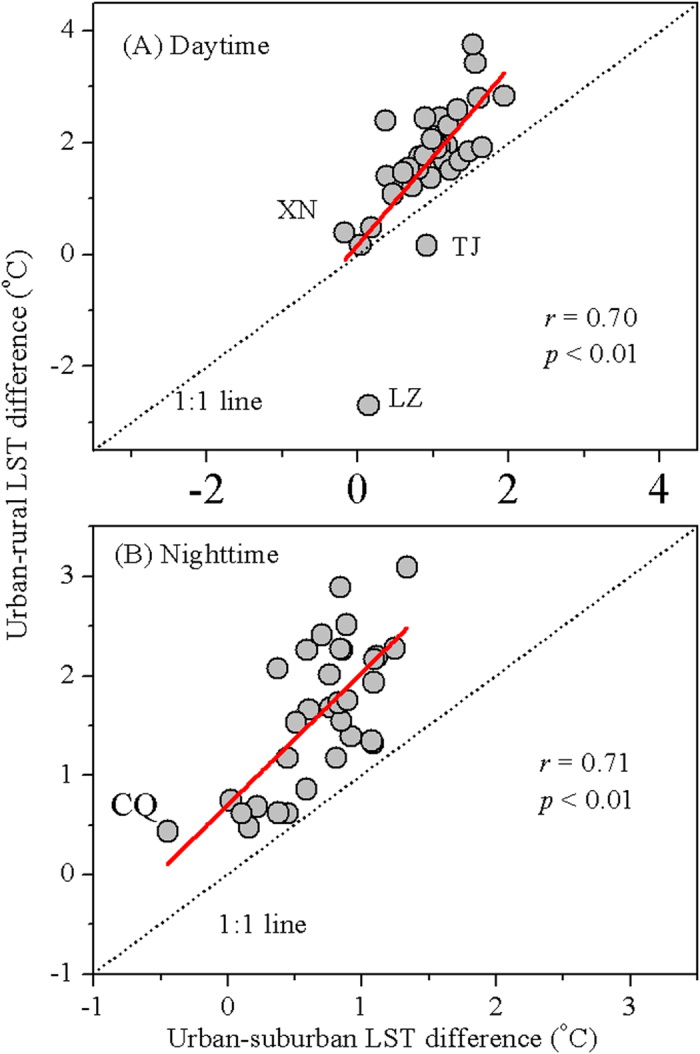
Relationship between annual mean urban-rural and urban-suburban LST differences averaged over 2003–2012 across China’s 32 major cities.

Emanating outward from urban edge to rural area, twelve buffers were generated for each map individually. The area of each buffer equals to half of the urban area (Fig. 2). Pixels that were water body or those with elevations more than 50 m higher than the highest point in the urban area were excluded from this analysis because these pixels may overshadow the urbanization effect on temperature^17^,^25^. We generated urban and buffer areas for each city map in 2005 and 2010. Since our timeframe was between 2003 and 2012, we assumed that the urban and buffer zones delineated in 2005 and 2010 can be used to represent those in 2003–2007 and 2008–2012, respectively.

In order to quantify the △T, the reference rural LST was first defined. We used the mid-value of the mean LST in the three farthest buffers as the rural LST for each city. This definition should be able to reflect the background LST because the distances of these buffers were obviously larger than the previously reported FP (i.e., less than 1.4 times of actual urban land size away from urban perimeter)^10^,^18^. The reason for using mid-value was to reduce the bias caused by the possible outlier among the three buffers. We calculated the LST differences (i.e., △T) in urban area and adjacent buffers for each city separately as follow:


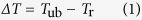


where *T*_*ub*_and *T*_*r*_ represent the LST over urban (or buffer) and reference rural areas, respectively. A positive value of △T indicates UHI effect, while the opposite means cold island effect. Then we estimated the mean △T over the period 2003–2012 and significance tests (*p* = 0.05) were conducted to determine if the mean △T was statistically different from zero (e.g., does there exist significant UHI effect?).

A single exponential decay model^18^ was used to examine the △T trends toward rural areas during the day and night time in different seasons (annual, summer, and winter) for each city and then the mean for all the cities. The formula takes the following form:


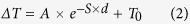


where *d* represents the distance away from the urban areas, *A* indicates the maximum temperature difference (close to UHII), *S* is the decay rate, and *T*_*0*_ is the asymptotic value that the exponential trend can reach (close to zero). The larger *S* means a shorter distance needed for the △T to level off given the same *A*, suggesting a smaller footprint of UHI effect (i.e., FP).

The FP represents areas significantly affected by UHI, including both urban and surrounding buffer areas. Since the △T varied substantially across space and time^10^,^11^,^12^, it is difficult to quantify the FP for all those cities using statistical models. Zhang et al.^18^ assumed that the FP can be determined based on the distance at which each of the exponential models reaches 95 percent of their asymptotic values (i.e., T_0_). However, this method failed to calculate the FP for the cities that had insignificant trends of the △T toward rural areas, in particular during the day in winter in this analysis. Therefore, we quantified the FP as the continuous extent emanating outward from urban centers to rural areas with the △T statistically larger than zero.

We estimated the FP in different time periods for each city separately. Then we assessed the impacts of UHII and actual urban size on the FP, hypothesizing that the city has a larger FP provided with a greater UHII or urban area. The UHII was defined as the △T in urban area in this analysis. We also explored the climatic effects (annual precipitation and mean air temperature) on the FP’s spatial variability. The Pearson’s correlations were estimated in SPSS PASW Statistics 18 (SPSS Inc.).

To address the possible UHII bias induced by methods ignoring the FP, we compared the UHII estimates under two most widely used definitions: urban-rural^17^,^21^ and urban-suburban LST differences^10^,^11^,^20^. Suburban area was defined as the adjacent buffer zone that covers the same area of urban land cover following previous publications^10^,^11^. The former estimate was assumed to have considered the FP, since the rural area defined in this study was far enough away from urban center that can reflect the background unaffected LST. The second was hypothesized to have ignored the FP, because the reference suburban area defined by us may have significant UHI effect^18^.

## Additional Information

**How to cite this article**: Zhou, D. *et al.* The footprint of urban heat island effect in China. *Sci. Rep.* **5**, 11160; doi: 10.1038/srep11160 (2015).

## Supplementary Material

Supplementary Information

## Figures and Tables

**Table 1:** Pearson’s correlations between the footprint of urban heat island effect (unit: times of urban area) and the potential drivers across China’s 32 major cities.

**Time periods**	**Temperature**	**Precipitation**	**UHII**
Summer day	−0.27	−0.35^b^	0.50^a^
Summer night	−0.36^b^	−0.35^b^	0.53^a^
Winter day	0.37^b^	0.48^a^	0.58^a^
Winter night	−0.63^a^	−0.59^a^	0.74^a^

UHII represents the urban heat island intensity that was defined as the temperature difference between urban and rural areas.

^a^significant at 0.01 level.

^b^significant at 0.05 level.
