# Supplementary material for: The footprint of urban heat island effect in China
Source: Sci Rep. 2015 Jun 10;5:11160. doi: 10.1038/srep11160 (PMC4461918; doi:10.1038/srep11160)
Supplement: Supplementary Information [file srep11160-s1.doc]

# The footprint of urban heat island effect in China

Decheng Zhou^1, 2^, Shuqing Zhao^2^, *, Liangxia Zhang^1^, Ge Sun^3^, Yongqiang Liu^4^

^1^ International Center for Ecology, Meteorology, and Environment (IceMe), and Jiangsu Key Laboratory of Agricultural Meteorology, Nanjing University of Information Science and Technology, Nanjing 210044, China.

^2^ College of Urban and Environmental Sciences, and Key Laboratory for Earth Surface Processes of the Ministry of Education, Peking University, Beijing 100871, China.

^3^ Eastern Forest Environmental Threat Assessment Center, Southern Research Station, USDA Forest Service, Raleigh, NC 27606, USA

^4^ Center for Forest Disturbance Science, Southern Research Station, USDA Forest Service, Athens, GA 30602, USA

**Correspondence to: S.Q. Zhao (**sqzhao@urban.pku.edu.cn**)**

## Supplementary information

**Table S1** The maximum temperature difference (*A*), decay rate (*S*), *r^2^*, and *p*-value for the exponential trends of the urban heat island effect (△T) with distance (*d*) away from urban centers to rural areas for China's 32 major cities averaged over 2003-2012. The function takes form of *△T* = *A*×e*^-S×d^*+*T*_0_, where T_0_ is the asymptotic value that the exponential trend can reach (close to zero and do not show in the table).

| City | *A* | *S* | *r^2^* | *p* | *A* | *S* | *r^2^* | *p* | *A* | *S* | *r^2^* | *p* |
| --- | --- | --- | --- | --- | --- | --- | --- | --- | --- | --- | --- | --- |
|  | Daytime annual | | | | Daytime summer | | | | Daytime winter | | | |
| Beijing | 2.00 | 1.07 | 0.95 | 0.00 | 4.40 | 1.04 | 0.98 | 0.00 | -0.07^a^ | 3.51^a^ | 0.04 | 0.83 |
| Changchun | 1.19 | 1.54 | 0.93 | 0.00 | 3.35 | 1.57 | 1.00 | 0.00 | 0.25 | 14.27^a^ | 0.42 | 0.07 |
| Changsha | 1.83 | 1.06 | 0.99 | 0.00 | 2.54 | 1.42 | 0.98 | 0.00 | 0.64 | 0.15 | 0.87 | 0.00 |
| Chengdu | 2.88 | 0.41 | 0.98 | 0.00 | 4.12 | 0.64 | 0.99 | 0.00 | 1.96 | 0.15^a^ | 0.95 | 0.00 |
| Chongqing | 1.85 | 0.87 | 0.97 | 0.00 | 3.12 | 0.65 | 0.98 | 0.00 | 0.23^a^ | 0.87^a^ | 0.16 | 0.42 |
| Fuzhou | 3.98 | 1.27 | 0.89 | 0.00 | 4.41 | 1.58 | 0.83 | 0.00 | 1.01 | 65.53 | 0.79 | 0.00 |
| Guangzhou | 1.37 | 1.92 | 0.96 | 0.00 | 1.10 | 4.26^a^ | 0.81 | 0.00 | 0.21 | 3.09^a^ | 0.62 | 0.01 |
| Guiyang | 1.83 | 1.75 | 0.75 | 0.00 | 3.26 | 1.48 | 0.89 | 0.00 | - | - | 0.33 | 0.14 |
| Haikou | 3.00 | 1.32 | 0.95 | 0.00 | 3.23 | 1.25 | 0.94 | 0.00 | 0.69 | 56.72 | 0.56 | 0.04 |
| Hangzhou | 1.93 | 2.68^a^ | 0.77 | 0.00 | 2.25 | 4.57^a^ | 0.71 | 0.00 | - | - | 0.31 | 0.16 |
| Harbin | 1.65 | 1.26 | 0.86 | 0.00 | 3.39 | 1.28 | 0.97 | 0.00 | 0.98 | 0.47 | 0.90 | 0.00 |
| Hefei | 2.43 | 0.87 | 0.99 | 0.00 | 3.69 | 1.21 | 0.99 | 0.00 | -0.14 | -0.29 | 0.67 | 0.00 |
| Hohhot | 0.33 | 1.24^a^ | 0.48 | 0.04 | 2.38 | 0.95 | 0.92 | 0.00 | 0.15^a^ | 1.02^a^ | 0.19 | 0.34 |
| Jinan | 1.77 | 1.12 | 0.98 | 0.00 | 2.54 | 1.39 | 0.99 | 0.00 | 0.29^a^ | 0.50^a^ | 0.31 | 0.16 |
| Kunming | 4.22 | 0.64 | 0.88 | 0.00 | 4.77 | 0.57 | 0.90 | 0.00 | 4.04^a^ | 0.10^a^ | 0.61 | 0.01 |
| Lanzhou | **11.91** | **-0.04** | **0.93** | **0.00** | **4.29** | **-0.10** | **0.89** | **0.00** | **10.90** | **-0.04** | **0.93** | **0.00** |
| Lhasa | 0.66^a^ | 0.09^a^ | 0.05 | 0.78 | 3.02 | 0.55^a^ | 0.76 | 0.00 | - | - | 0.13 | 0.42 |
| Nanchang | 1.60 | 1.08 | 0.94 | 0.00 | 1.97 | 1.08 | 0.97 | 0.00 | - | - | 0.22 | 0.30 |
| Nanjing | 1.55 | 2.39 | 0.89 | 0.00 | 1.82 | 3.03 | 0.84 | 0.00 | - | - | 0.18 | 0.37 |
| Nanning | 2.19 | 1.21 | 0.93 | 0.00 | 2.53 | 0.76 | 0.93 | 0.00 | 0.29^a^ | 1.81^a^ | 0.15 | 0.46 |
| Shanghai | 3.09 | 1.65 | 0.97 | 0.00 | 3.37 | 2.52 | 0.90 | 0.00 | 0.63 | 0.56 | 0.84 | 0.00 |
| Shenyang | 1.68 | 2.48 | 0.98 | 0.00 | 3.65 | 1.96 | 0.99 | 0.00 | - | - | 0.62 | 0.01 |
| Shenzhen | 2.40 | 0.87 | 0.92 | 0.00 | 2.37 | 0.87 | 0.90 | 0.00 | 1.60^a^ | 0.13^a^ | 0.61 | 0.01 |
| Shijiazhuang | 1.43 | 0.55 | 0.96 | 0.00 | 2.77 | 1.30 | 0.99 | 0.00 | - | - | 0.58 | 0.01 |
| Taiyuan | 1.30 | 1.08 | 0.92 | 0.00 | 2.70 | 1.15 | 0.98 | 0.00 | - | - | 0.33 | 0.14 |
| Tianjin | - | - | 0.20 | 0.33 | 2.05 | 2.59 | 0.92 | 0.00 | **-43.47** | **0.01** | **0.96** | **0.00** |
| Urumqi | 1.66 | 0.99 | 0.95 | 0.00 | 2.10 | 0.48 | 0.95 | 0.00 | 0.53 | 1.25^a^ | 0.67 | 0.00 |
| Wuhan | 2.38 | 2.01^a^ | 0.72 | 0.00 | 3.41 | 2.13 | 0.81 | 0.00 | - | - | 0.49 | 0.03 |
| Xi'an | 2.02 | 1.30 | 0.92 | 0.00 | 1.72 | 2.78 | 0.92 | 0.00 | - | - | 0.10 | 0.60 |
| Xining | 0.85 | 0.54^a^ | 0.52 | 0.03 | 2.33 | 0.41 | 0.89 | 0.00 | - | - | 0.19 | 0.35 |
| Yinchuan | 2.77 | 0.79 | 0.93 | 0.00 | 6.77 | 0.82 | 0.97 | 0.00 | 0.34^a^ | 0.33^a^ | 0.23 | 0.27 |
| Zhengzhou | 2.73 | 1.04 | 0.99 | 0.00 | 4.18 | 1.15 | 0.98 | 0.00 | 0.14^a^ | 1.16^a^ | 0.04 | 0.84 |
|  | Nighttime annual | | | | Nighttime summer | | | | Nighttime winter | | | |
| Beijing | 2.09 | 1.02 | 0.99 | 0.00 | 2.03 | 1.21 | 1.00 | 0.00 | 1.33 | 0.57 | 0.90 | 0.00 |
| Changchun | 1.30 | 2.65 | 0.99 | 0.00 | 0.95 | 3.06 | 0.92 | 0.00 | 0.75 | 0.28 | 0.93 | 0.00 |
| Changsha | 1.60 | 1.16 | 0.99 | 0.00 | 1.37 | 1.16 | 0.98 | 0.00 | 0.62 | 1.11 | 0.88 | 0.00 |
| Chengdu | 1.26 | 1.52 | 0.95 | 0.00 | 1.30 | 1.16 | 0.98 | 0.00 | 0.90^a^ | 0.20^a^ | 0.55 | 0.02 |
| Chongqing | 0.80 | 0.30 | 0.46 | 0.00 | 1.52 | 1.11 | 0.99 | 0.00 | 0.69 | 9.66^a^ | 0.41 | 0.00 |
| Fuzhou | 3.02 | 0.18^a^ | 0.92 | 0.00 | 2.29 | 0.35 | 0.93 | 0.00 | -0.74 | -0.17 | 0.88 | 0.00 |
| Guangzhou | 0.71 | 1.60 | 0.86 | 0.00 | 0.62 | 1.62 | 0.76 | 0.00 | 0.35 | 1.70^a^ | 0.55 | 0.02 |
| Guiyang | 1.65 | 0.40 | 0.92 | 0.00 | 1.40 | 0.46 | 0.91 | 0.00 | -0.60 | -0.14 | 0.51 | 0.01 |
| Haikou | 1.84 | 0.34 | 0.95 | 0.00 | 2.33 | 0.18^a^ | 0.92 | 0.00 | 2.04 | 0.59 | 0.95 | 0.00 |
| Hangzhou | 0.75 | 0.70 | 0.81 | 0.00 | 1.02 | 0.34^a^ | 0.88 | 0.00 | 0.65 | 0.86 | 0.83 | 0.00 |
| Harbin | 1.65 | 0.52 | 0.97 | 0.00 | 1.12 | 0.41 | 0.93 | 0.00 | 1.71 | 0.47 | 0.97 | 0.00 |
| Hefei | 0.56 | 2.49 | 0.81 | 0.00 | 0.46 | 1.07 | 0.96 | 0.00 | - | - | 0.05 | 0.78 |
| Hohhot | 2.76 | 0.47 | 0.99 | 0.00 | 2.38 | 0.54 | 0.99 | 0.00 | 3.26 | 0.23 | 0.97 | 0.00 |
| Jinan | 1.75 | 0.60 | 0.97 | 0.00 | 1.48 | 0.47 | 0.96 | 0.00 | 1.24 | 0.46 | 0.93 | 0.00 |
| Kunming | 2.19 | 0.63^a^ | 0.70 | 0.00 | 3.55 | 0.61 | 0.78 | 0.00 | 1.09^a^ | 0.60^a^ | 0.31 | 0.16 |
| Lanzhou | 3.24 | 0.34 | 0.99 | 0.00 | 2.61 | 0.51 | 0.98 | 0.00 | 5.21 | 0.14 | 0.98 | 0.00 |
| Lhasa | 2.61 | 0.35 | 0.97 | 0.00 | 2.99 | 0.48 | 0.96 | 0.00 | 3.16 | 0.12^a^ | 0.97 | 0.00 |
| Nanchang | 1.30 | 0.65 | 0.98 | 0.00 | 1.12 | 0.94 | 0.94 | 0.00 | 1.15 | 0.35 | 0.92 | 0.00 |
| Nanjing | 1.04 | 0.41 | 0.90 | 0.00 | 0.78 | 0.60 | 0.89 | 0.00 | 1.47 | 0.21 | 0.89 | 0.00 |
| Nanning | 1.42 | 1.62 | 0.98 | 0.00 | 1.07 | 1.86 | 0.98 | 0.00 | 0.45 | 3.42^a^ | 0.49 | 0.04 |
| Shanghai | 0.50 | 0.80 | 0.92 | 0.00 | 0.52 | 1.23 | 0.85 | 0.00 | 0.62 | 0.33 | 0.92 | 0.00 |
| Shenyang | 1.40 | 2.13 | 0.98 | 0.00 | 1.34 | 1.76 | 0.99 | 0.00 | 0.53 | 3.75^a^ | 0.64 | 0.01 |
| Shenzhen | 0.97 | 1.69 | 0.87 | 0.00 | 1.19 | 0.86 | 0.88 | 0.00 | 0.34^a^ | 1.63^a^ | 0.21 | 0.31 |
| Shijiazhuang | 1.94 | 1.10 | 1.00 | 0.00 | 1.68 | 1.31 | 0.99 | 0.00 | 1.25 | 0.65 | 0.96 | 0.00 |
| Taiyuan | 2.12 | 0.65 | 1.00 | 0.00 | 2.06 | 0.77 | 0.99 | 0.00 | 1.45 | 0.39 | 0.97 | 0.00 |
| Tianjin | 2.30 | 0.63 | 1.00 | 0.00 | 1.77 | 0.67 | 0.99 | 0.00 | 1.99 | 0.40 | 0.97 | 0.00 |
| Urumqi | 3.10 | 0.76 | 0.99 | 0.00 | 3.81 | 0.69 | 0.99 | 0.00 | 1.60 | 0.69 | 0.87 | 0.00 |
| Wuhan | 0.72 | 0.14 | 0.80 | 0.00 | 0.42 | 0.71 | 0.95 | 0.00 | - | - | 0.21 | 0.30 |
| Xi'an | 1.52 | 0.68 | 0.93 | 0.00 | 1.49 | 1.07 | 0.93 | 0.00 | -0.69 | -0.15 | 0.92 | 0.00 |
| Xining | 2.58 | 0.62 | 0.98 | 0.00 | 2.61 | 0.59 | 0.98 | 0.00 | 1.97 | 0.60 | 0.97 | 0.00 |
| Yinchuan | 2.20 | 1.17 | 0.98 | 0.00 | 2.07 | 1.24 | 0.97 | 0.00 | 1.28 | 0.87 | 0.97 | 0.00 |
| Zhengzhou | 2.07 | 0.90 | 0.94 | 0.00 | 1.69 | 0.96 | 0.99 | 0.00 | 1.64 | 0.32^a^ | 0.66 | 0.00 |

^a^, the estimated coefficient was not significant at 0.05 level (T-test);

-, insignificant exponential decay trend and has abnormal value;

The bold records indicate that there were significant exponential trends of cold island effect along urban-rural gradients.

**Table S2** Landsat images for land use classification used in this study.

| Cities | path/row (WRS2) | Period (Year-mouth-day) | | Cities | path/row (WRS2) | Period (Year-mouth-day) | |
| --- | --- | --- | --- | --- | --- | --- | --- |
|  |  | 2005 | 2010 |  |  | 2005 | 2010 |
| Beijing | 123/32 | 2005-7-25 | 2010-8-8 | Lanzhou | 131/35 | 2006-8-5 | 2010-8-24 |
|  | 123/33 | 2005-7-25 | 2010-8-8 |  | 130/35 | 2006-10-17 | 2010-10-4 |
| Changchun | 118/29 | 2005-9-8 | 2010-9-22 |  | 131/34 | 2006-8-5 | 2009-7-28 |
|  | 118/30 | 2005-9-18 | 2010-6-2 | Lhasa | 137/39 | 2005-11-16 | 2009-10-26 |
| Changsha | 123/40 | 2005-5-6 | 2010-9-17 |  | 137/40 | 2005-11-16 | 2009-10-26 |
|  | 123/41 | 2005-5-6 | 2010-9-17 |  | 138/38 | 2006-5-18 | 2008-6-8 |
|  | 124/40 | 2005-7-16 | 2009-6-17 |  | 138/39 | 2004-1-20 | 2010-2-6 |
|  | 124/41 | 2005-7-16 | 2007-9-16 |  | 138/40 | 2004-1-20 | 2010-2-6 |
| Chengdu | 129/39 | 2005-3-5 | 2010-3-19 | Nanchang | 121/40 | 2004-10-4 | 2010-10-5 |
|  | 130/38 | 2005-4-13 | 2010-3-18 | Nanjing | 120/37 | 2005-9-16 | 2010-9-9 |
|  | 130/39 | 2006-5-2 | 2010-3-18 |  | 120/38 | 2005-9-16 | 2010-5-20 |
| Chongqing | 127/39 | 2006-8-9 | 2010-8-12 | Nanning | 125/43 | 2006-9-12 | 2010-11-2 |
|  | 127/40 | 2004-8-3 | 2010-10-31 |  | 125/44 | 2006-9-12 | 2010-11-2 |
|  | 128/39 | 2006-9-1 | 2010-9-20 |  | 125/45 | 2006-9-12 | 2010-11-2 |
|  | 128/40 | 2004-9-1 | 2010-9-20 |  | 126/43 | 2004-12-2 | 2010-10-24 |
| Fuzhou | 119/42 | 2006-11-5 | 2010-11-8 |  | 126/44 | 2004-12-2 | 2010-10-24 |
| Guangzhou | 122/43 | 2005-11-23 | 2010-10-28 | Shanghai | 118/38 | 2005-8-15 | 2009-7-17 |
|  | 122/44 | 2005-11-23 | 2010-10-28 |  | 118/39 | 2005-8-15 | 2009-7-17 |
| Guiyang | 127/41 | 2005-4-8 | 2009-5-21 | Shenyang | 119/30 | 2006-9-18 | 2010-9-29 |
|  | 127/42 | 2005-4-8 | 2010-10-31 |  | 119/31 | 2006-9-18 | 2010-9-29 |
| Haikou | 123/46 | 2004-6-28 | 2010-7-31 | Shenzhen | 121/44 | 2004-10-12 | 2009-10-18 |
|  | 124/46 | 2004-12-20 | 2010-2-28 |  | 122/44 | 2005-11-23 | 2010-10-28 |
| Hangzhou | 119/39 | 2005-10-17 | 2010-8-20 | Shijiazhuang | 124/33 | 2005-6-22 | 2010-7-6 |
|  | 119/40 | 2005-10-17 | 2010-3-21 |  | 124/34 | 2005-6-22 | 2010-7-6 |
|  | 120/39 | 2006-5-20 | 2009-6-5 | Taiyuan | 125/33 | 2006-8-27 | 2010-9-23 |
|  | 120/40 | 2006-5-20 | 2009-6-5 |  | 125/34 | 2006-8-27 | 2010-9-23 |
| Harbin | 116/28 | 2006-9-29 | 2010-9-8 |  | 126/34 | 2006-6-15 | 2010-7-12 |
|  | 116/29 | 2006-9-13 | 2010-9-8 | Tianjin | 122/32 | 2005-8-27 | 2010-10-28 |
|  | 117/28 | 2006-9-20 | 2010-9-15 |  | 122/33 | 2005-10-14 | 2010-9-10 |
|  | 117/29 | 2006-9-20 | 2010-9-15 | Urumqi | 142/30 | 2006-9-3 | 2010-8-13 |
|  | 118/28 | 2006-9-27 | 2010-9-22 |  | 143/30 | 2006-6-22 | 2009-7-16 |
|  | 118/29 | 2006-9-27 | 2010-9-22 | Wuhan | 122/39 | 2005-7-18 | 2010-10-28 |
| Hefei | 121/37 | 2005-8-12 | 2010-10-5 |  | 123/38 | 2005-9-11 | 2010-9-17 |
|  | 121/38 | 2005-8-12 | 2010-3-11 |  | 123/39 | 2005-9-11 | 2010-9-17 |
| Hohhot | 126/32 | 2006-6-15 | 2009-7-9 | Xi'an | 127/36 | 2004-6-24 | 2010-8-28 |
|  | 127/31 | 2006-9-10 | 2010-9-5 |  | 127/37 | 2004-6-8 | 2010-8-28 |
|  | 127/32 | 2006-9-10 | 2010-9-5 |  | 126/36 | 2005-9-8 | 2010-10-8 |
| Jinan | 122/34 | 2005-9-4 | 2009-8-30 | Xining | 132/34 | 2006-8-4 | 2010-7-30 |
|  | 122/35 | 2006-5-2 | 2009-8-30 |  | 132/35 | 2004-9-15 | 2010-7-30 |
| Kunming | 129/42 | 2005-4-6 | 2011-4-15 | Yinchuan | 129/33 | 2005-5-8 | 2010-9-11 |
|  | 129/43 | 2006-4-1 | 2010-1-30 |  | 129/34 | 2005-5-24 | 2010-9-11 |
|  | 130/42 | 2005-5-15 | 2011-4-6 | Zhengzhou | 124/36 | 2004-8-30 | 2009-6-25 |
|  | 130/43 | 2004-12-14 | 2011-4-6 |  |  |  |  |
